# Warfarin–Linezolid Interaction: A Case of Severe Coagulopathy

**DOI:** 10.1155/crdi/5132611

**Published:** 2025-12-11

**Authors:** Aniqa Batool, Waqar Khan, Muhammad Mohsin

**Affiliations:** ^1^ National Institute of Cardiovascular Diseases, Karachi, Pakistan, nicvd.gov.bd

**Keywords:** atrial fibrillation, drug–drug interaction, infective endocarditis, linezolid, warfarin

## Abstract

**Background:**

Warfarin, a vitamin K antagonist, is commonly used for atrial fibrillation (AF), venous thromboembolism (VTE), and mechanical heart valves. Linezolid, an oxazolidinone antibiotic, is used to treat severe infections caused by Gram‐positive bacteria. A fatal drug interaction of linezolid and warfarin was reported in this case, highlighting the close monitoring of international normalized ratio (INR) when co‐administering these drugs.

**Case Presentation:**

A 62‐year‐old female with severe mitral stenosis (MS) and moderate mitral regurgitation (MR) was diagnosed with mitral valve vancomycin‐resistant *Enterococcus* (VRE) infective endocarditis (IE) (the diagnosis was made based on clinical presentation, positive blood cultures, and echocardiographic evidence fulfilling the modified Duke criteria) AF, and acute kidney injury (AKI). The AKI was resolved during the hospital stay. The patient was on the valvular surgery list and was discharged on oral linezolid and warfarin. The patient was on follow‐up compliance with restricted diet through INR online services, and INR was also monitored through the well‐established INR clinic. Ten days postdischarge, she presented with worsening dyspnea, bruising, hematuria, abdominal distension, and bilateral leg swelling. On admission, she was on rapid AF, severe metabolic acidosis, hyperkalemia, and coagulopathy (INR 12). Given her adherence to warfarin and the absence of other interacting factors, the coagulopathy was suspected to be due to a warfarin–linezolid interaction. Despite management with IV vitamin K and fresh frozen plasma (FFP), she developed refractory AKI, hyperkalemia, and multiorgan failure, leading to death.

**Conclusion:**

This case highlights the potential for severe coagulopathy when warfarin and linezolid are co‐administered and underscores the importance of close and frequent INR monitoring.

## 1. Introduction

Warfarin, a vitamin K antagonist, is widely indicated for the treatment of atrial fibrillation (AF), venous thromboembolism (VTE), and mechanical heart valves [1]. Its narrow therapeutic index necessitates frequent international normalized ratio (INR) monitoring due to numerous drug and food interactions that affect its metabolism [2]. Linezolid is an oxazolidinone antibiotic used to treat severe Gram‐positive infections, including those caused by multidrug‐resistant pathogens [3, 4]. Its co‐administration with warfarin may enhance the anticoagulation effect, requiring careful monitoring [5, 6]. This case report presents a severe coagulopathy potentially linked to a warfarin–linezolid interaction.

## 2. Case Presentation

A 62‐year‐old female with a history of severe mitral stenosis (MS) and moderate mitral regurgitation (MR) was hospitalized with the diagnosis of mitral valve infective endocarditis (IE) caused by vancomycin‐resistant *Enterococcus* (VRE), AF, and acute kidney injury (AKI). Echocardiography revealed severe left and right atrial dilation, a D‐shaped left ventricle with an ejection fraction (EF) of 40%, and thickened, domed mitral valves. Significant findings included severe MS, moderate‐to‐severe MR, and severe tricuspid regurgitation. The AKI was resolved during the hospital stay. The patient was discharged without any clinical evidence of infection on a regimen that included oral linezolid 600 mg every 12 h for 4 weeks, warfarin 5 mg once daily, and bisoprolol 5 mg once daily. After discharge, she was monitored in an INR clinic for warfarin therapeutic management through telehealth services every two to 3 days and for diet restriction compliance to avoid any potential cause that leads to increased INR. The patient’s INR was between 2.5 and 3.3 as monitored through INR clinic.

**Figure 1:**
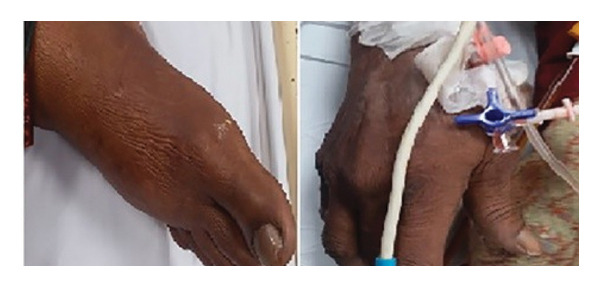
Clinical presentations of bruises in the emergency department with an INR of 12.

Ten days after discharge, she presented to the emergency department (ED) with extensive bruising, hematuria, worsening dyspnea, abdominal distension, bilateral leg swelling, and decreased appetite. The clinical presentation of bruises is shown in Figure 1. On admission, she was tachycardic (HR 100–130 bpm) with a blood pressure of 110/69 mmHg and an oxygen saturation of 94%. An electrocardiogram (ECG) confirmed AF, while arterial blood gas (ABG) analysis showed severe metabolic acidosis and hyperkalemia. A coagulation profile revealed a critically elevated INR of 12. The laboratory values during the first and second hospital stay are illustrated in Table 1. Given the patient’s adherence to warfarin, the absence of dietary or other drug interactions, and the recent initiation of linezolid, the coagulopathy was suspected to be due to a warfarin–linezolid interaction. The warfarin was discontinued, and the patient’s conditions were managed in the following manner.

**Table 1:** Laboratory values during hospitalization.

Laboratory parameters	First‐time admission							Readmission				
	Day 1	Day 2	Day 3	Day 4	Day 5	Day 6	Day 7	Day 0	Day 1	Day 2	Day 3	Day 4
INR	1.95	1.51	1.88	1.59	2.09	3	3	12	2.6	2.65	2	3.92
Cr. (mg/dL)	2	1.61	1.4	1.4	0.91	0.7	0.6	1.8	2.11	3.61	4.5	4.3
K (mEq/)	3.7	3.5	3	2.3	3.1	2.9	2.6	5.1	5.6	6		7
Procalcitonin (ng/mL)								—	2.98	—		
Hs‐CRP (mg/dL)								3.53	3.6	—		4.06
Trop. I (ng/L)											42.2	
Urea (mg/dL)								94.2	104.9	—	203	206
Hg (g/dL)	12.8	12.3	11	11	11	12	11.8	8.9	9.2	8.9	12	12.8
RBC (k/μL)	4.29	4.08	3.92	4.19	3.98	4.32	4.11	3.11	3.19	3.1	4.27	4.26
Platelets (K/μL)	152	134	106	107	122	137	145	67	77	27	275	159
WBC (K/μL)	6.19	5.94	8.9	8.3	6.19	7.35	8.1	3.79	4.3	2.1	6.4	6.2
ESR								20				10
Bilirubin (g/mL)	1.95	1.49	1.53	1.94	1.44	1.56	—					
Gamma (U/L)	60	67	64	71	64	80	—					
SGPT (ALT) (U/L)	19	19	20	25	25	25	—					
SGOT (AST) (U/L)	77	60	64	56	78	40	—					
AP (U/L)	129	112	79	87	76	100	—					

To manage the coagulopathy, the patient was treated with intravenous vitamin K (10 mg), and six fresh frozen plasma (FFP) units as per clinical guidelines. While prothrombin complex concentrate (PCC) was considered, it was not available at our facility at the time of management [7]. Despite these interventions, she rapidly deteriorated, developing cardiogenic shock, rapid AF (HR 168 bpm), persistent AKI, and refractory hyperkalemia. The progressive multiorgan failure ultimately led to her death. The clinical timeline from first admission, discharge, and readmission is illustrated in Table 2.

**Table 2:** Clinical timeline of events.

Hospital stay	Event/intervention	Key findings/outcome
Days 0–5 (first admission)	Diagnosed with vancomycin‐resistant *Enterococcus* (VRE) infective endocarditis, atrial fibrillation, and AKI	Started IV linezolid 600 mg q12 h and warfarin 5 mg daily
Days 6–8	Clinical improvement	Blood cultures became negative, and inflammatory markers improved
Day 8 (discharge)	Discharged home on oral linezolid + warfarin	INR 2.0, renal function improving
Day 3 postdischarge	Telehealth INR follow‐up	INR 3.1
Day 6 postdischarge	Continued telehealth monitoring	INR 3.2
Day 10 postdischarge (readmission)	Presented with bruising, hematuria, dyspnea	INR 12, AKI relapse, hyperkalemia
Days 11–14	Managed with IV vitamin K and FFP	INR decreased, but the patient developed multiorgan failure
Day 15 (outcome)	Cardiac arrest and death	Cause: severe coagulopathy secondary to warfarin–linezolid interaction

## 3. Discussion

This case highlights a potential interaction between warfarin and linezolid, leading to critical coagulopathy. Despite appropriate warfarin dosing and the absence of other known interacting factors, the patient developed a markedly elevated INR (12), progressing to coagulopathy and hemorrhagic complications. This case highlights the importance of close INR monitoring and early recognition of potential interactions when co‐administering linezolid and warfarin for a long course of regimen.

VRE endocarditis posed a therapeutic challenge due to limited antibiotic options and the risk of persistent bacteremia. Linezolid was chosen as the primary treatment, but its concomitant use with warfarin introduced a potential drug interaction, complicating anticoagulation management. While the warfarin–linezolid interaction was a likely contributor to the supratherapeutic INR in this case, linezolid may also potentiate warfarin’s effect via CYP450‐independent mechanisms, such as plasma protein–binding displacement and hepatic metabolic modulation. Although not a direct CYP450 inhibitor, linezolid has been implicated in enhancing warfarin anticoagulation through these alternative pathways, which may have contributed to the severe and refractory coagulopathy observed, especially amid multiorgan dysfunction [8].

Warfarin–linezolid interaction provides the most plausible explanation for the severe INR elevation; other potential contributors were considered, like hepatic dysfunction, malnutrition, and systemic inflammation [8]. In our case, all three contributing factors were unlikely, as liver enzymes and bilirubin levels remained within normal limits throughout hospitalization. Malnutrition and poor dietary vitamin K intake were ruled out based on caregiver reports confirming adequate oral intake after discharge. Similarly, systemic inflammation or uncontrolled infection was improbable, as the patient’s infection was clinically stable at the time of discharge and inflammatory markers (CRP, procalcitonin). Therefore, the coagulopathy was most consistent with a pharmacologic interaction between warfarin and linezolid.

The potential mechanism of this interaction may involve multiple pathways. Linezolid is known to suppress intestinal microbiota responsible for vitamin K synthesis, ultimately enhancing the anticoagulant effect of warfarin. This indirect mechanism likely amplified warfarin’s pharmacologic action in this patient. Moreover, linezolid has been independently associated with hematologic abnormalities, such as thrombocytopenia and elevated INR, particularly in patients with renal impairment/clinically unstable/shock conditions [9]. However, given the patient’s stable hepatic function, absence of malnutrition, and temporal relationship between the initiation of linezolid and INR elevation, the most plausible explanation remains a pharmacodynamic interaction between warfarin and linezolid, resulting in excessive anticoagulation.

Enterococcal bacteremia has been associated with disseminated intravascular coagulation (DIC) [10]; classical DIC was unlikely in this patient. Apart from an elevated INR and transient thrombocytopenia, there was no evidence of progressive platelet decline, hypofibrinogenemia, or markedly elevated D‐dimer levels. The infection was clinically controlled at discharge, and there were no signs of ongoing sepsis. Thus, the temporal association and laboratory pattern strongly suggest that the coagulopathy resulted primarily from a warfarin–linezolid interaction.

In this report, the critically elevated INR (12), coagulopathy, and hematuria were likely consequences of this linezolid and warfarin interaction. The patient’s condition further deteriorated due to cardiogenic shock and AKI, which led to multiorgan dysfunction and mortality.

## 4. Conclusion

To conclude, this report highlights the potential for a clinically significant interaction between warfarin and linezolid, leading to life‐threatening coagulopathy. Given the absence of other contributing factors, this interaction should be considered when managing patients on warfarin who require linezolid therapy. Close and frequent INR monitoring, early recognition of coagulopathy, and careful anticoagulation adjustments are essential in preventing adverse outcomes, especially when this prescription is essential for a long period of time.

## Ethics Statement

This case report was conducted in accordance with institutional ethical standards and the principles of the Declaration of Helsinki. Formal ethical approval was waived as this is a single‐patient case report with no identifiable information. Written informed consent was obtained from the patient’s next of kin for publication of this case and any accompanying clinical information.

## Conflicts of Interest

The authors declare no conflicts of interest.

## Author Contributions

All authors contributed to the study’s conception, data analysis, and manuscript preparation.

## Funding

This research did not receive any specific grant from funding agencies in the public, commercial, or not‐for‐profit sectors.

## Data Availability

All data supporting the findings of this case report are included within the article. Further data will be provided upon request.
